# Plasma proteome changes in cardiovascular disease patients: novel isoforms of apolipoprotein A1

**DOI:** 10.1186/1479-5876-9-84

**Published:** 2011-06-01

**Authors:** Pavel Májek, Zuzana Reicheltová, Jiří Suttnar, Martin Malý, Milan Oravec, Klára Pečánková, Jan E Dyr

**Affiliations:** 1 Institute of Hematology and Blood Transfusion, Prague, Czech Republic; 2 2nd Faculty of Medicine, Dept. of Cardiology, Charles University, Prague, Czech Republic

## Abstract

**Background:**

The aim of this proteomic study was to look for changes taking place in plasma proteomes of patients with acute myocardial infarction (AMI), unstable angina pectoris (UAP), and stable angina pectoris (SAP).

**Methods:**

Depleted plasma proteins were separated by 2D SDS-PAGE (pI 4-7), and proteomes were compared using Progenesis SameSpots statistical software. Proteins were identified by nanoLC-MS/MS. Proteins were quantified using commercial kits. Apolipoprotein A1 was studied using 1D and 2D SDS-PAGE, together with western blotting.

**Results:**

Reciprocal comparison revealed 46 unique, significantly different spots; proteins in 34 spots were successfully identified and corresponded to 38 different proteins. Discrete comparisons of patient groups showed 45, 41, and 8 significantly different spots when AMI, UAP, and SAP were compared with the control group. On the basis of our proteomic data, plasma levels of two of them, alpha-1 microglobulin and vitamin D-binding protein, were determined. The data, however, failed to prove the proteins to be suitable markers or risk factors in the studied groups. The plasma level and isoform representation of apolipoprotein A1 were also estimated. Using 1D and 2D SDS-PAGE, together with western blotting, we observed extra high-molecular weight apolipoprotein A1 fractions presented only in the patient groups, indicating that the novel high-molecular weight isoforms of apolipoprotein A1 may be potential new markers or possible risk factors of cardiovascular disease.

**Conclusion:**

The reported data show plasma proteome changes in patients with AMI, UAP, and SAP. We propose some apolipoprotein A1 fractions as a possible new disease-associated marker of cardiovascular disorders.

## Introduction

Cardiovascular disease (CVD) is the major cause of premature death in Europe. It is an important cause of decrease in quality of life, disability, and contributes substantially to the escalating costs of health care [1]. Generally, the epidemic of CVD is a global phenomenon, and the magnitude of its increase in incidence has potentially major implications for countries that represent much of the developed world. There are two major approaches to the prevention of CVD: public health/community-based strategies and clinical-based strategies with a targeted approach to high-risk patients using modern methods to estimate risk factors, plus various combinations of these approaches. Thus, laboratory medicine now plays a crucial role in identifying risk factors, early events, and conditions triggering plaque rupture in coronary heart disease [2,3]. The greatest progress in laboratory research has resulted in the discovery of new and more-promising biochemical markers of myocardial damage [4].

Nowadays modern scientific techniques like genomics, proteomics, and metabolomics serve to discover new therapeutic targets and biomarkers [5,6]. In this way newly achieved insights into the mechanisms of cardiovascular disease can lead to proposals of new modern therapies and to the improvement of CVD diagnostics and therapy monitoring [7]. The importance of measuring proteins as biomarkers has become increasingly clear, as mRNA transcripts cannot be directly correlated to protein expression [8]; and posttranslational modifications are known to be instrumental in many human diseases, including CVD.

Although the cardiac myocytes are the best source that might provide the possibility to observe proteome changes in the failed heart tissue, plasma protein biomarkers have been the focus of extensive study in recent years [4,6,9,10]. Moreover, cardiac myocytes are not usually available for diagnoses purposes; and blood collection is a quick and simple procedure that is less invasive and easily executable, with almost no patient discomfort.

Proteomics offers a combination of different techniques to analyze proteins in a sample at a given time with the detection of protein levels, isoforms, posttranslational modifications, etc. The possibility of exploring the current state of organism, tissues, or other subproteomes of interest (cells, plasma, etc.) is an essential attribute of proteomics. Information obtained by comparing proteomic results between different groups or under different conditions may be useful in general, as well as at a personalized patient level [11].

The aim of this study was to compare plasma proteomes of patients with acute myocardial infarction (AMI), unstable angina pectoris (UAP), stable angina pectoris (SAP), and control subjects to find any significant protein differences within the groups of study, and to search for potential new proteomic markers of CVD.

## Methods

A total of 130 patient plasma samples were used in this proteomic study. The samples were divided into four individual groups: patients with acute myocardial infarction with ST elevations; patients with unstable angina pectoris; patients with stable angina pectoris; and a control group of donors exhibiting a normal coronary angiogram. Characteristics of the study groups are summarized in Table 1.

**Table 1:** Characteristics of the study groups

	AMI (n = 27)	UAP (n = 26)	SAP (n = 38)	Control (n = 39)
Age (years ± SEM)	65 ± 14	72 ± 12	66 ± 10	67 ± 12
Males n (%)	16 (59)	18 (69)	28 (74)	14 (36)
Hypertension n (%)	12 (44)	23 (88)	34 (89)	28 (72)
Dyslipidemia n (%)	14 (52)	16 (62)	32 (84)	26 (67)
Diabetes n (%)	6 (22)	7 (27)	15 (39)	8 (21)
Smoking n (%)	13 (48)	5 (19)	7 (18)	4 (10)
History of MI n	2	5	12	2
History of stroke n	0	4	4	1
BMI	28.3 ± 3.3	27.9 ± 4.3	28.7 ± 5.3	27.5 ± 4.4
creatinine (μmol/L)	87.7 ± 31.6	88.5 ± 26.5	87.5 ± 26.9	75.3 ± 17.6
treatment n (%):				
Aspirin	5 (19)	14 (54)	26 (68)	20 (51)
ADP blockers	1 (4)	6 (23)	12 (32)	6 (15)
Beta blockers	4 (15)	10 (39)	25 (66)	20 (51)
ACE inhibitors	2 (7)	16 (62)	22 (58)	12 (31)
ARB	2 (7)	1 (4)	5 (13)	8 (21)
Statins	2 (7)	11 (42)	28 (74)	20 (51)
Ca blockers	1 (4)	5 (19)	11 (29)	6 (15)
Warfarin	2 (7)	2 (8)	4 (11)	2 (5)

*AMI *acute myocardial infarction, *SAP *stable angina pectoris, *UAP *unstable angina pectoris, smoking reflects current smokers, body-mass index (BMI) and serum creatinine are expressed as average values ± standard deviation

All acute coronary syndrome patients were classified according to criteria including chest pain, ECG changes (ST elevation, non ST elevation) and troponin I positivity. All these patients underwent coronary angiography - patients with ST elevation myocardial infarction immediately in the regime of primary PCI, and patients without ST elevations within 24 hours of admission with PCI of culprit lesion.

All tested individuals agreed to participate in this study, gave informed consent, and the study was approved by the Ethics Committee of the Motol University Hospital. All samples were obtained and analyzed in accordance with the Ethical Committee regulations of the Institute of Hematology and Blood Transfusion. All investigations conform to the Declaration of Helsinki.

Blood was collected by venipuncture into tubes coated with EDTA within the 24-hour window with respect to cardiac events. Plasma was obtained by the centrifugation (15 min, 3000 × g) of blood samples; and plasma aliquots were then transferred to polypropylene Eppendorf tubes and stored at -70°C until used.

Thawed plasma samples were centrifuged (5 min, 12000 × g), diluted 1:4 by depletion buffer (Agilent, Santa Clara, CA, USA), and filtrated using 0.22 μm Spin filters (Agilent) for 1 minute by centrifugation at 12000 × g. A MARS Hu-6 4.6 × 100 mm column (Agilent) was used to remove six high-abundant proteins (albumin, IgG, α1-antitrypsin, IgA, transferrin, and haptoglobin). 5K MWCO Spin Concentrators (Agilent) were used to desalinate and concentrate the samples (3000 × g, 20°C). MilliQ water (4 mL) was added to concentrated samples; and the desalinating-concentrating step was repeated three times. Finally, desalinated and concentrated samples were vacuum dried, frozen rapidly, and stored at -70°C.

1D electrophoresis was performed using 10 μL of an undepleted plasma sample that was resuspended in 200 μL of reducing sample buffer (62.5 mM Tris, 4.05% w/v SDS, 20% v/v glycerol, 10% v/v 2-mercaptoethanol, and a trace of bromophenol blue). The sample was then heated at 95°C for 3 min, centrifuged; and 10 μL of the sample was used for analysis. Proteins were separated by SDS-PAGE (8 × 10 cm, 10% gradient gel, 3.75% stacking gel, 5°C, 30 mA/gel) using a P8SD vertical electrophoresis separation system (Owl; Thermo Scientific, Waltham, MA, USA).

2D SDS-PAGE, image analysis, protein digestion, and mass spectrometry analysis were performed as described before [12]. Briefly, isoelectric focusing (IPG strips pI 4-7, 7.7 cm) and SDS-PAGE (conditions as used in 1D SDS-PAGE) were used in the first and in the second dimension, respectively. Gels were stained with colloidal Coomassie blue, scanned, and processed with Progenesis SameSpots software (Nonlinear Dynamics, Newcastle upon Tyne, UK). All four groups (AMI, UAP, SAP, and normal control group - N) were compared with each other; and fold values, as well as p-values of all spots, were computed with the above mentioned software, using one way ANOVA analysis. Moreover, separate comparisons of each patient group relative to the control group were created. PCA (Principal Component Analysis) of the compared groups was performed by the SameSpot software, using the spots of statistical significance. Protein identification was then only conducted on spots that significantly differed. The selected spots were excised from the gel, and proteins were in-gel digested by trypsin. An HCT ultra ion-trap mass spectrometer (Bruker Daltonics, Bremen, Germany) with nanoelectrospray ionization, coupled to a UltiMate 3000 nanoLC system (Dionex, Sunnyvale, CA, USA) was used to perform MS analysis. MASCOT (Matrix Science, London, UK) was used for database searching (SWISS-PROT release 2010_12). Two unique peptides (fulfilling a minimal Mascot score) were necessary to successfully identify a protein.

Western blotting was performed using an Owl HEP-1 Semi Dry Electroblotting System (Thermo Scientific). Proteins were transferred from gel to a PVDF membrane (10 V constant voltage for 1 hr); the membrane was then incubated with a blocking buffer (3% BSA in PBS) at 4°C overnight, rinsed; and incubated with goat anti-Apolipoprotein A1 horseradish peroxidase conjugated antibody (Baria, Psary, Czech Republic), 1:10000 dilution, at 37°C for 30 min. After rinsing, a chemiluminescent substrate (SuperSignal West Pico; Thermo Scientific) was added to the membrane for 5 min; and a 2 hr film exposition (Amersham Hyperfilm ECL; GE Life Sciences, Piscataway, NJ, US) was performed. After which, the film was developed and scanned.

Apolipoprotein A1 (apoA1), alpha-1 microglobulin (a1m), and vitamin D-binding protein (VDBP) plasma levels were measured using kits (ApoA1 assay KAI-002 and Apo A1/B calibrator KAI-008C, Alpha-1 Microglobulin assay KAI-056 and KAI-068C calibrator, and Vitamin D Binding Protein ELISA KT-515; all kits were purchased from Kamiya Biomedical, Seattle, WA, USA) according to manufacturer instructions. ApoA1 and a1m were measured in triplet, VDBP in duplicate. Results were expressed as means ± standard deviations. One-way ANOVA was used to determine the statistical significance of plasma level changes of apoA1 and a1m in samples, while an unpaired t-test was used for the comparison of VDBP when the AMI group was compared with the control group.

## Results

2D gels were prepared for the experiments, and scanned gel images were divided into four individual groups according to patient group disposal: acute myocardial infarction (AMI, n = 27), unstable angina pectoris (UAP, n = 26) stable angina pectoris (SAP, n = 38), and normal control (N, n = 39). All four groups were compared together, with a separate comparison of individual groups relative to the control group.

**Figure 1:**
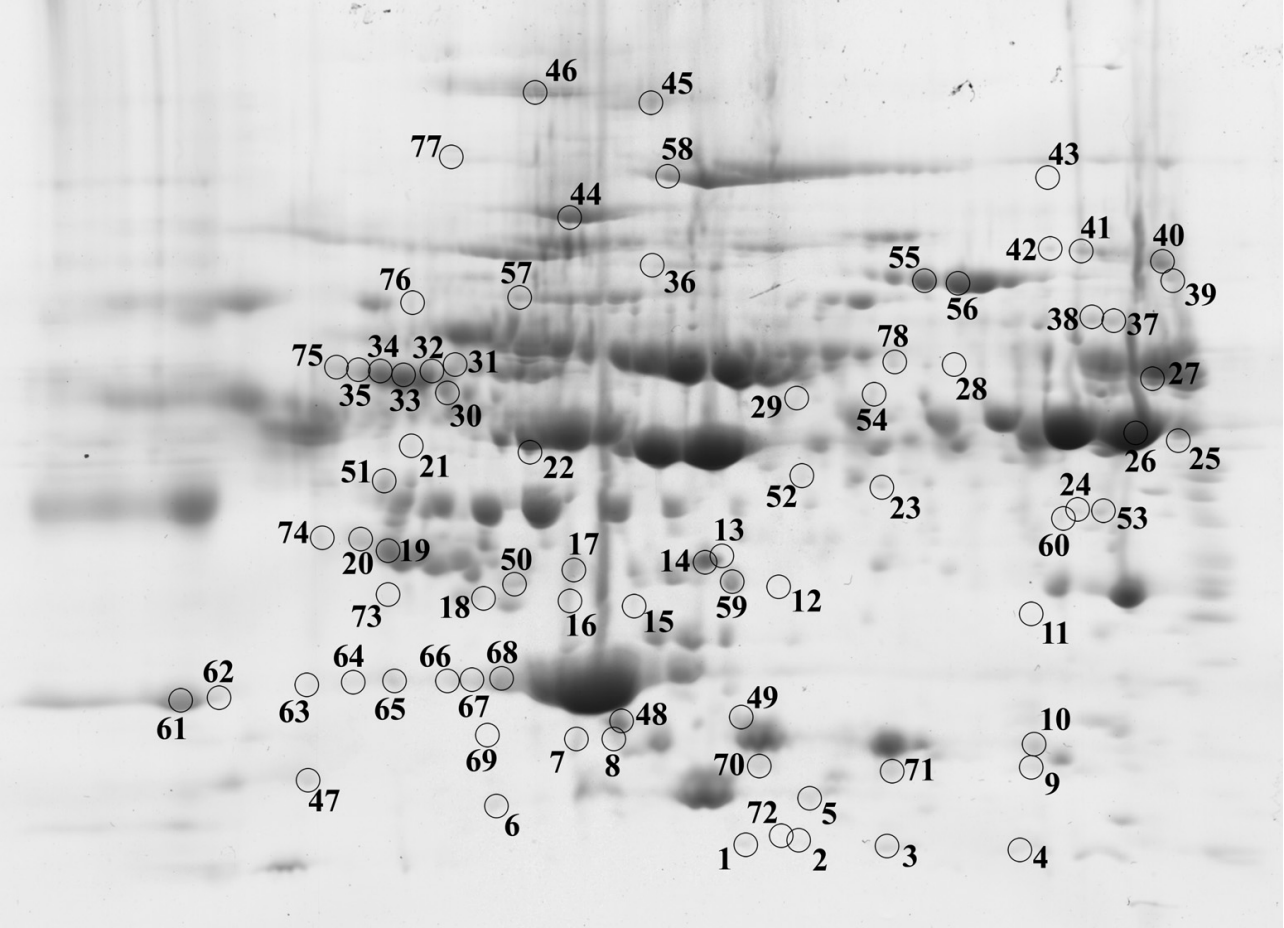
**Positions of significantly differed spots on a 2D gel**. Positions of all spots that were found to significantly differ in 2D gels of reciprocal comparison of all four groups and separate comparisons of the three patient groups with the control group.

Reciprocal comparison of all four groups revealed 46 unique spots that significantly (p < 0.05) differed in normalized volumes (Figure 1); the proteins in 34 of the 46 spots were successfully identified by mass spectrometry. These 34 identified spots correspond to 38 different proteins. The list of spots, including protein identification, sequence coverage (%), protein accession number, fold value, ANOVA p-value, and logarithms of spot normalized volume (LSNV) with its standard deviation for each group, is summarized in (Additional file 1: Table S1). The fold value shows the fold difference between the groups with the lowest and highest normalized volumes.

Separately comparing the three patient groups with the control group we found: 45 significantly differing spots when comparing AMI with N (Figure 1), 44 of which were successfully identified and corresponded to 38 different proteins; 41 significantly differing spots when comparing UAP with N (Figure 1), 38 of which were successfully identified and corresponded to 23 different proteins; 8 significantly differing spots when comparing SAP with N (Figure 1), 5 of which were successfully identified and corresponded to 9 different proteins. The lists of spots, including protein identification, sequence coverage (%), protein accession number, fold value, and ANOVA p-value, are summarized in (Additional file 2: Table S2). The fold value shows the fold difference of distinct groups relative to the control group.

**Figure 2:**
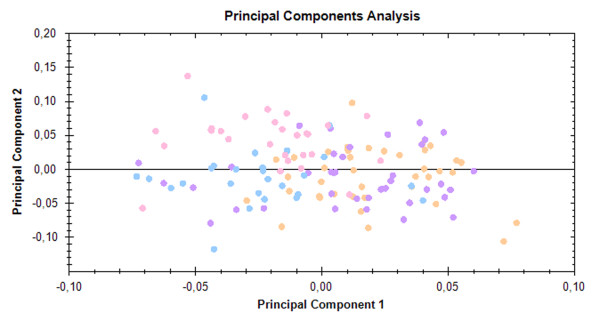
**Principal Component Analysis (PCA) of all compared groups**. PCA shows separation into three aggregates using the first two principal components. Acute coronary syndrome group composed of AMI (pink) and UAP (blue) is distinguishable according to the Principle Component 1. According to the Principle Component 2 the three PCA resolved groups of samples are composed of mutually resolved AMI and UAP samples, and of a group comprising SAP (violet) and control samples (yellow).

PCA of the mutual comparison of all four groups indicated the distinct separation of AMI, UAP, and a third aggregate that contained SAP and the control group (Figure 2). According to the Principle Component 1, there are two aggregates: the first composed of acute coronary syndrome groups (AMI and UAP) and the second containing SAP and the control group. When the Principle Component 2 is taken into account, it is apparent that the acute coronary syndrome group is separated into two discrete groups (AMI and UAP) (Figure 2).

Plasma levels of three different proteins (apolipoprotein A1, alpha-1 microglobulin, and vitamin D-binding protein) were quantified. Plasma concentrations of apoA1 (mg/dL) were determined in each respective group: 124.1 ± 17.3 (AMI, n = 28); 118.0 ± 23.0 (UAP, n = 28); 132.5 ± 28.0 (SAP, n = 27); and 147.2 ± 28.2 (N, n = 34). The statistical analysis showed a significant difference between the groups (ANOVA p = 0.000055). Plasma concentrations of a1m (mg/L) were determined as follows: 25.0 ± 6.0 (AMI, n = 36); 24.9 ± 6.5 (UAP, n = 34); 26.8 ± 7.5 (SAP, n = 40); and 22.2 ± 5.9 (N, n = 37). A significant difference between the groups (ANOVA p = 0.0369) was also observed. VDBP plasma concentration (ug/mL) was determined in control and AMI groups: 327.0 ± 52.4 (AMI, n = 20) and 327.3 ± 52.0 (N, n = 20). No significant difference was observed between these two groups.

**Figure 3:**
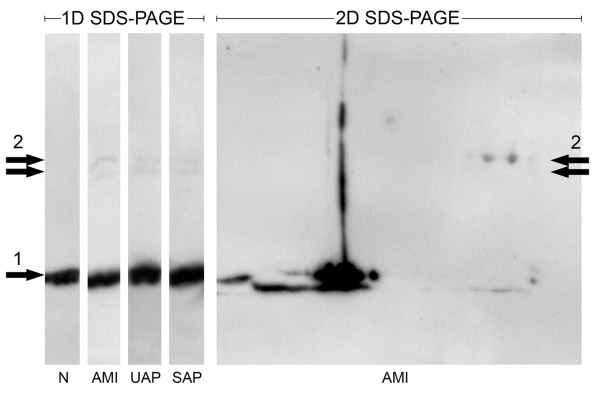
**Apolipoprotein A1 isoforms pattern**. Representation of apolipoprotein A1 (apoA1) regular band (1) and extra high MW fraction (2) in all four groups (N - control group, AMI - acute myocardial infarction, UAP - unstable angina pectoris, and SAP - stable angina pectoris) using 1D and 2D western blotting.

Western blot analysis of 1D gels (20 samples of each group) was performed to observe apoA1 fractions. In all patient group samples (AMI, UAP, SAP) extra fractions of higher molecular weight (MW) were observed (in addition to the regular apoA1 fraction); whereas no such extra fractions were observed in the control group (Figure 3). 2D western blot analysis was then performed, and several extra spots (with MW corresponding to those of 1D extra apoA1 bands) were observed. Protein identification confirmed the presence of apoA1 only in one fourth of all identified samples.

## Discussion

Many of the identified proteins are known markers, risk factors, or proteins known to be involved in cardiovascular disease; e.g. transthyretin, apoA1, fibrinogen, serum amyloid A protein, haptoglobin, plasminogen, and others [2,6]. Our study presents both a comparison of the respective patient groups with a control group, and the mutual comparison of all groups together; and thus offers deeper insight into proteome changes. As an example, spot 26 (fibrinogen alpha and beta chains) significantly differed in AMI vs. N and UAP vs. N, whereas no significant difference was found in SAP vs. N. The results obtained from the mutual group comparison show that the LSNV value of spot 26 is also increased in SAP (with an approximate 50% increase when compared to those in AMI and UAP); and the changes observed in acute coronary syndrome (AMI and UAP) are non-significantly present in SAP. Fibrinogen chains are known to be involved in cardiovascular disease (gamma chain variants associate with increased thrombotic risk; alpha and beta chains have been found to be modified; etc.) [13,14].

We have also found several new possible protein biomarker candidates: lumican, protein AMBP, VDBP, and biotinidase. They have not been previously associated with CVD; or their role in CVD is not known. On the basis of our data, we further focused on two proteins, quantifiable with commercially available immunodetection kits, to estimate their plasma levels and thus to consider their roles as possible biomarkers - VDBP and protein AMBP. In apoA1, a known marker of CVD, we assessed its plasma levels, including proportional representation of isoforms.

Vitamin D-binding protein is a multifunctional protein, which carries vitamin D sterols, associates with membrane-bound immunoglobulin on the surface of B-lymphocytes, prevents the polymeratization of actin by binding actin monomers, etc. As a part of the actin scavenging system, VDBP binds actin released from cells upon injury; wherein these complexes are then rapidly cleared from the bloodstream [15]. It is assumed that the influence of VDBP prevents the harmful effects of actin filaments in blood vessels. VDBP is also considered to be a regulator of the inhibitory effect of acetylsalicylic acid on platelets [16]. We observed VDBP spot change within acute coronary syndrome groups (AMI and UAP) exclusively in the AMI group; therefore VDBP plasma level in AMI was compared with the control group. We did not, however, observe any significant VDBP level difference within these groups. So, the observed spot change might be caused either by posttranslational modification of VDBP in the AMI group or by other co-identified proteins in the spot.

Protein AMBP is a complex glycoprotein secreted in plasma. Its precursor is proteolytically processed into distinct functioning proteins: alpha-1-microglobulin (a1m), a low molecular weight plasma glycoprotein, and an inter-alpha-trypsin inhibitor light chain (bikunin) that is further cleaved to trypstatin [17]. All protein AMBP peptides identified by mass spectrometry were part of the a1m sequence (peptides: 44-57, 63-85, 167-185, and 186-202; the amino acid sequence of protein AMBP). Moreover, molecular mass estimated by SDS-PAGE corresponded to a1m. The function and physiological role of a1m is not fully understood; nevertheless, this protein presumably plays a role in the regulation of the immune system, and is involved in defending tissue against oxidation by reactive oxygen species and heme [18]. A1m is known to be complexed to macromolecules (immunoglobulin A, albumin, and prothrombin), as well as to be present in a free form of a monomeric protein [18]. This protein is considered to be a marker of renal insufficiency, and to reflect the overall inflammatory status in patients with arterial hypertension and normal renal function [19]. We found that spot 18 (protein AMBP) differed significantly when mutually comparing all four groups. LSNV values of this spot were decreased in all three patient groups with the lowest value in UAP. The comparison of individual groups with the control group showed another significant LSNV decrease (UAP vs. N, spot 18, p = 0.002) and showed a difference in spot 50 (AMI vs. N) with non-unique protein identification. We quantified a1m plasma levels within all four groups to compare them with the results obtained by 2D-electrophoresis. We found significant differences of a1m plasma concentrations; nevertheless, the results did not correspond to those observed by 2D-electrophoresis. This is probably caused by posttranslational modification (UAP vs. N, spot 18) or by the presence of other proteins in spot 50 (AMI vs. N), as in the case of the VDBP described above. Summarizing our results, a1m does not seem to be a suitable marker or even a risk factor of cardiovascular diseases.

ApoA1 participates in the transport of cholesterol (between tissues and the liver) by promoting cholesterol efflux and by acting as a cofactor for the lecithin cholesterol acyltransferase. This protein is known to be a useful tool in the assessment of coronary heart disease risk. High levels of apoA1 can help to prevent heart disease, and it has also been shown that apoA1 plasma level is decreased in cardiovascular disease [2]. Further, it has been demonstrated that several apoA1 isoforms are present in plasma, and that at least some of these isoforms probably change during acute coronary syndrome [20]. Our 2D SDS-PAGE results showed significant changes in apoA1 LSNV values (all patient groups vs. N, AMI vs. N, UAP vs. N), as well as changes of several isoforms (spots 61-69, UAP vs. N). We quantified apoA1 plasma levels in all four groups to assess whether the changes are based on apoA1 isoforms (thus confirming the role of posttranslational modifications) or on a total concentration. ApoA1 plasma levels were significantly decreased as compared with the control in all three patient groups, with the minimum in UAP in agreement with previous literature [21-23]. The majority of apoA1 changes obtained by 2D SDS-PAGE were observed in UAP vs. N (10 spots, 9 of them unique identification). This is consistent with the highest concentration difference (UAP vs. N) obtained by apoA1 quantification. If apoA1 changes were based on plasma concentration only, the values of both differences (in plasma concentration and that observed in 2D electrophoresis) should be the same. ApoA1 plasma level change in UAP vs. N was found to be approximately 20%, whereas apoA1 isoforms LSNV changes (spots 61-69) range from 20% to 80%. This indicates that apoA1 changes in UAP are based not only on plasma concentration differences, but also on posttranslational changes of apoA1 isoforms. We performed western blotting with immunodetection to observe all spots in 2D SDS-PAGE, which could contain apoA1 to test the possibility of the influence of non-identified apoA1 spots to the results. The spots observed by immunodetection matched those observed in 2D SDS-PAGE in which apoA1 was identified. Surprisingly, we also detected a region with a group of several apoA1 spots of higher molecular weight (approximately 50 kDa) than that of apoA1 (28 kDa). Based on this finding, 1D SDS-PAGE of 20 samples of each group (AMI, UAP, SAP, and N) followed by immunoblotting, was performed to estimate the possible presence of the high MW apoA1 fractions in other groups. Two different extra bands with detected apoA1 were observed (in addition to the regular apoA1 bands) in all samples of all patient groups (AMI, UAP, and SAP), while no extra bands were detected in the samples of the control group (Figure 3). Despite the low number of replicates (n = 20, each group) and the observed variability of band intensities, we assume that the high MW apoA1 fraction might be a new disease-associated marker of cardiovascular disorders. The non-significant change of this fraction in 2D SDS-PAGE analysis might be caused by a low concentration not detectable by 2D electrophoresis, or by overlapping with more abundant proteins in spots. Finally, several 2D gels, as well as 2D western blots, were prepared to estimate the pattern of the high MW apoA1 fraction spots, and to identify the extra apoA1 fraction by mass spectrometry. The spot pattern in 2D gels was extremely variable as expected, and corresponded to bands observed in the 1D gels. We assume that either apoA1 is covalently linked (due to the denaturing conditions of SDS-PAGE) to another protein, or that apoA1 fragments are linked to a protein or proteins. Unfortunately, mass spectrometry analysis did not reveal the composition of the extra apoA1 fraction, with only a quarter of all identified samples containing apoA1. This might be caused by the presence of high-abundant proteins that overlap the spots as observed by western blotting. The presence of apoA1 fragments instead of the intact protein molecule could be another explanation. It has been shown that there are apoa1 fragments present in the plasma of patients with nephrotic syndrome [24].

The Principal Component Analysis applied to the data obtained within all four groups of samples showed their separation into three rather well resolved aggregates using the first two principal components (Figure 2). CVD patients in this study can be stratified by traditional clinical diagnoses into acute syndrome groups (AMI together with UAP) and into SAP. This stratification (one acute coronary syndrome group composed of AMI and UAP) is clearly distinguishable according to the Principle Component 1. According to the Principle Component 2, the three well-resolved PCA groups of samples are composed of mutually resolved AMI and UAP samples and of a group comprising SAP and control samples. The SAP and control samples are poorly distinguished mutually, using a given relatively small set of proteins for PCA, as compared with the normal healthy population without atherosclerosis. This may be due to comparable age and the common presence of risk factors in both SAP and control patients. The obtained results of the PCA method are thus in agreement with the stratification of patients using classical clinical diagnostics.

The role of new markers is not only to distinguish the studied groups. The knowledge of protein markers and changes in their concentration and/or posttranslation modification should increase our understanding of the mechanisms that are involved in coronary disease, may constitute new potential risk factors and help to predict personal risk and thus help in the treatment. The current markers (troponin etc.) are sufficient to help or predict diagnosis and distinguish the groups used in this study. However, the role of novel apoA1 isoforms as a new risk factor should be further investigated. It is not known at present whether the apoA1 isoforms are in patients' plasma as a result or the cause of the patient's disease. Moreover, it is hard to speculate on the role and possible involvement of apoA1 isoforms in cardiovascular diseases: to date neither the composition of these isoforms, nor the influence of other factors (age, treatment, smoking, etc.) on the level and composition of these isoforms is known.

## Conclusion

The reported data show plasma proteome changes in patients with acute myocardial infarction, unstable angina pectoris, and stable angina pectoris. Proteins that are known markers or risk factors of CVD were observed, as well as new possible marker candidates. On the basis of our data, we tested two of the candidates, vitamin D-binding protein and alpha-1 microglobulin, as potential CVD markers. The estimation of their plasma levels, however, did not indicate them as suitable CVD markers. The testing of the known CVD marker, apoA1, revealed the possible role of biomarker isoforms and their posttranslational modifications. Therefore, we propose the high MW apoA1 fraction as a possible new disease-associated marker of cardiovascular disorders.

## Competing interests

The authors declare that they have no competing interests.

## Authors' contributions

PM, ZR and JS designed and performed research, analyzed data and wrote the manuscript. MM and MO collected samples and designed research. KP performed research and analyzed data. JED designed research and wrote the manuscript. All authors read and approved the final manuscript.

## Supplementary Material

Additional file 1 **Table S1**. List of spots that significantly differ in plasma proteomes of patients with acute myocardial infarction, unstable angina pectoris, and stable angina pectoris. Click here for file

Additional file 2 **Table S2**. Lists of spots that significantly differ when separately comparing the three patient groups with the control group. Click here for file
