# Supplementary material for: Plasma proteome changes in cardiovascular disease patients: novel isoforms of apolipoprotein A1
Source: J Transl Med. 2011 Jun 1;9:84. doi: 10.1186/1479-5876-9-84 (PMC3224581; doi:10.1186/1479-5876-9-84)
Supplement: Additional file 1 — Table S1. List of spots that significantly differ in plasma proteomes of patients with acute myocardial infarction, unstable angina pectoris, and stable angina pectoris. [file 1479-5876-9-84-S1.PDF]

Additional file 1: Table S1 List of spots that significantly differ in plasma proteomes of patients with acute myocardial infarction, unstable angina pectoris, and stable angina pectoris.

						log of spot normalized volume			
**spot**	**protein**	**sc %**	**ac**	**fold**	**p**	**N**	**SAP**	**UAP**	**AMI**
1	Serum amyloid A protein	52	P02735	2.5	0.008	6.95 ± 0.13	6.94 ± 0.16	7.19 ± 0.24	7.1 ± 0.2
2	unidentified			1.4	0.011	6.61 ± 0.08	6.67 ± 0.08	6.75 ± 0.1	6.64 ± 0.09
3	Serum amyloid A protein	52	P02735	2.4	0.005	7.29 ± 0.09	7.3 ± 0.12	7.49 ± 0.21	7.49 ± 0.23
4	Serum amyloid A protein	40	P02735	1.6	0.014	6.87 ± 0.13	6.95 ± 0.1	7.04 ± 0.15	6.83 ± 0.15
5	unidentified			1.4	0.011	6.85 ± 0.09	6.91 ± 0.08	6.91 ± 0.1	6.79 ± 0.09
6	Transthyretin	30	P02766	1.3	0.049	6.88 ± 0.08	6.85 ± 0.08	6.81 ± 0.09	6.77 ± 0.08
7	Transthyretin	55	P02766	1.4	0.033	7.06 ± 0.11	6.93 ± 0.1	6.92 ± 0.11	6.93 ± 0.16
8	Transthyretin	39	P02766	1.4	0.018	7.44 ± 0.09	7.36 ± 0.1	7.33 ± 0.1	7.29 ± 0.13
9	unidentified			1.8	0.046	6.34 ± 0.11	6.34 ± 0.13	6.42 ± 0.18	6.22 ± 0.12
10	unidentified			1.4	0.042	6.82 ± 0.11	6.88 ± 0.13	6.97 ± 0.12	6.8 ± 0.14
11	Ig kappa chain C region	48	P01834	1.3	0.017	6.33 ± 0.08	6.35 ± 0.06	6.27 ± 0.1	6.24 ± 0.09
12	Apolipoprotein E	9	P02649	1.9	0.01	6.63 ± 0.09	6.7 ± 0.11	6.63 ± 0.08	6.8 ± 0.18
13	Transthyretin	55	P02766	1.5	0.008	7 ± 0.13	6.93 ± 0.14	6.78 ± 0.19	6.81 ± 0.17
14	Transthyretin	64	P02766	1.3	0.005	8.13 ± 0.05	8.12 ± 0.07	8.04 ± 0.12	7.99 ± 0.15
15	Apolipoprotein A-I	12	P02647	1.5	0.04	7.16 ± 0.09	7.1 ± 0.11	7.08 ± 0.14	7.01 ± 0.1
16	Transthyretin	55	P02766	1.7	0.034	7.34 ± 0.12	7.26 ± 0.12	7.18 ± 0.09	7.27 ± 0.11
	Apolipoprotein A-I	16	P02647						
17	Apolipoprotein A-I	26	P02647	1.5	0.026	7.51 ± 0.11	7.42 ± 0.12	7.36 ± 0.1	7.39 ± 0.09
	Inter-alpha-trypsin inhibitor heavy chain H4	7	Q14624						
	Apolipoprotein E	13	P02649						
18	Protein AMBP	11	P02760	1.4	0.035	6.49 ± 0.08	6.44 ± 0.09	6.37 ± 0.07	6.42 ± 0.09
19	Clusterin	27	P10909	1.2	0.005	8 ± 0.04	8 ± 0.05	7.93 ± 0.05	7.97 ± 0.05
20	unidentified			1.2	0.024	7.48 ± 0.05	7.45 ± 0.07	7.38 ± 0.06	7.41 ± 0.08
21	unidentified			1.3	0.033	6.65 ± 0.07	6.56 ± 0.07	6.55 ± 0.09	6.58 ± 0.1
22	Fibrinogen gamma chain	43	P02679	1.2	0.018	7.87 ± 0.07	7.95 ± 0.05	7.94 ± 0.09	7.96 ± 0.07
	Antithrombin-III	12	P01008						
	Vitamin D-binding protein	13	P02774						
	Apolipoprotein A-IV	16	P06727						
23	unidentified			1.5	0.02	6.46 ± 0.13	6.46 ± 0.12	6.52 ± 0.14	6.31 ± 0.15
24	unidentified			1.6	0.033	6.16 ± 0.11	6.17 ± 0.13	6.23 ± 0.16	6.04 ± 0.13
25	Fibrinogen beta chain	33	P02675	1.8	0.019	6.79 ± 0.13	6.7 ± 0.12	6.93 ± 0.2	6.85 ± 0.18
	Plasma serine protease inhibitor	20	P05154						
	Fibrinogen gamma chain	25	P02679						
	Carboxypeptidase N catalytic chain	6	P15169						
26	Fibrinogen beta chain	50	P02675	1.2	0.045	8.92 ± 0.06	8.95 ± 0.04	8.99 ± 0.07	8.99 ± 0.05
	Fibrinogen alpha chain	6	P02671						
27	Complement C3	25	P01024	1.2	0.017	8.5 ± 0.07	8.51 ± 0.07	8.58 ± 0.06	8.57 ± 0.06
	Fibrinogen alpha chain	21	P02671						
	Fibrinogen beta chain	23	P02675						
28	Serum albumin	33	P02768	1.7	0.027	7.43 ± 0.16	7.31 ± 0.15	7.24 ± 0.19	7.21 ± 0.16
	Fibrinogen alpha chain	7	P02671						
	Hemopexin	19	P02790						
29	Hemopexin	23	P02790	3.4	0.014	6.71 ± 0.22	6.58 ± 0.21	6.66 ± 0.23	6.4 ± 0.11
	Alpha-2-macroglobulin	5	P01023						
	Serum albumin	9	P02768						
30	Kininogen-1	15	P01042	1.3	0.02	7.29 ± 0.07	7.34 ± 0.06	7.22 ± 0.1	7.27 ± 0.09
	Angiotensinogen	12	P01019						
	Thyroxine-binding globulin	10	P05543						
31	Alpha-2-antiplasmin	25	P08697	1.2	0.034	7.11 ± 0.05	7.1 ± 0.06	7.04 ± 0.08	7.12 ± 0.05
	Complement component C9	4	P02748						
	Histidine-rich glycoprotein	8	P04196						
32	Vitronectin	8	P04004	1.4	0.006	6.93 ± 0.1	6.94 ± 0.09	6.8 ± 0.11	6.83 ± 0.1
	Kininogen-1	8	P01042						
33	Vitronectin	8	P04004	1.4	0.0008	7.86 ± 0.06	7.87 ± 0.09	7.74 ± 0.1	7.76 ± 0.09
	Kininogen-1	8	P01042						
	Lumican	20	P51884						
34	Vitronectin	8	P04004	1.4	0.0001	7.74 ± 0.07	7.75 ± 0.08	7.59 ± 0.1	7.65 ± 0.08
	Kininogen-1	8	P01042						
35	unidentified			1.4	0.0004	7.34 ± 0.07	7.34 ± 0.09	7.18 ± 0.1	7.25 ± 0.08
36	Fibrinogen gamma chain	32	P02679	1.6	0.005	6.79 ± 0.1	6.79 ± 0.09	6.81 ± 0.15	6.97 ± 0.13
	Plasma protease C1 inhibitor	8	P05155						
	Hemopexin	12	P02790						
37	unidentified			1.6	0.014	7.22 ± 0.12	7.3 ± 0.12	7.19 ± 0.12	7.11 ± 0.13
38	unidentified			1.6	0.006	6.87 ± 0.12	6.92 ± 0.13	6.78 ± 0.12	6.71 ± 0.15
39	Complement factor B	16	P00751	1.6	0.017	6.76 ± 0.11	6.73 ± 0.14	6.81 ± 0.16	6.94 ± 0.16
	Complement C4-A; Complement C4-B	4; 4	P0C0L4; P0C0L5						
40	Plasminogen	31	P00747	1.4	0.002	7.63 ± 0.08	7.58 ± 0.12	7.67 ± 0.1	7.76 ± 0.09
41	Fibrinogen beta chain	30	P02675	1.4	0.012	7.28 ± 0.12	7.27 ± 0.1	7.29 ± 0.13	7.43 ± 0.1
	Plasminogen	25	P00747						
42	Beta-2-glycoprotein 1	24	P02749	1.4	0.03	6.98 ± 0.13	7.11 ± 0.11	7.07 ± 0.15	7.16 ± 0.13
	Complement C3	6	P01024						
43	unidentified			1.9	0.023	6.79 ± 0.12	6.96 ± 0.15	6.96 ± 0.2	7 ± 0.19
44	Ceruloplasmin	26	P00450	1.2	0.042	8.34 ± 0.06	8.28 ± 0.07	8.35 ± 0.06	8.36 ± 0.07
	Vitamin D-binding protein	27	P02774						
45	Fibronectin	10	P02751	1.4	0.022	7.36 ± 0.13	7.42 ± 0.12	7.48 ± 0.11	7.53 ± 0.11
	Fibrinogen gamma chain	22	P02679						
46	Ceruloplasmin	14	P00450	1.4	0.012	7.62 ± 0.12	7.62 ± 0.12	7.73 ± 0.15	7.79 ± 0.12
	Alpha-1B-glycoprotein	22	P04217						
	Inter-alpha-trypsin inhibitor heavy chain H2	5	P19823						

ac accession number (Swiss-Prot), *AMI* acute myocardial infarction, *N* control group, *SAP* stable angina pectoris, *sc %* protein sequence coverage, *UAP* unstable angina pectoris
