# Supplementary material for: Plasma proteome changes in cardiovascular disease patients: novel isoforms of apolipoprotein A1
Source: J Transl Med. 2011 Jun 1;9:84. doi: 10.1186/1479-5876-9-84 (PMC3224581; doi:10.1186/1479-5876-9-84)
Supplement: Additional file 2 — Table S2. Lists of spots that significantly differ when separately comparing the three patient groups with the control group. [file 1479-5876-9-84-S2.PDF]

Additional file 2: Table S2 Lists of spots that significantly differ when separately comparing the three patient groups with the control group.

List of spots that significantly differ when comparing plasma proteomes of acute myocardial infarction patients with the control group

spot	protein	sc %	ac	fold	p
3	Serum amyloid A protein	52	P02735	2.4	0.007
6	Transthyretin	30	P02766	-1.3	0.009
7	Transthyretin	55	P02766	-1.2	0.035
8	Transthyretin	39	P02766	-1.4	0.004
9	unidentified			-1.3	0.046
11	Ig kappa chain C region	48	P01834	-1.3	0.037
12	Apolipoprotein E	9	P02649	1.7	0.007
13	Transthyretin	55	P02766	-1.5	0.008
14	Transthyretin	64	P02766	-1.3	0.005
15	Apolipoprotein A-I	12	P02647	-1.5	0.002
17	Apolipoprotein A-I<br>Inter-alpha-trypsin inhibitor heavy chain H4<br>Apolipoprotein E	26<br>7<br>13	P02647<br>Q14624<br>P02649	-1.4	0.023
20	unidentified			-1.1	0.041
22	Fibrinogen gamma chain<br>Antithrombin-III<br>Vitamin D-binding protein<br>Apolipoprotein A-IV	43<br>12<br>13<br>16	P02679<br>P01008<br>P02774<br>P06727	1.2	0.01
23	unidentified			-1.4	0.023
24	unidentified			-1.3	0.03
26	Fibrinogen beta chain<br>Fibrinogen alpha chain	50<br>6	P02675<br>P02671	1.1	0.031
27	Complement C3<br>Fibrinogen alpha chain<br>Fibrinogen beta chain	25<br>21<br>23	P01024<br>P02671<br>P02675	1.2	0.036
28	Serum albumin<br>Fibrinogen alpha chain<br>Hemopexin	33<br>7<br>19	P02768<br>P02671<br>P02790	-1.7	0.007
29	Hemopexin<br>Alpha-2-macroglobulin<br>Serum albumin	23<br>5<br>9	P02790<br>P01023<br>P02768	-3.4	0.001
32	Vitronectin<br>Kininogen-1	8<br>8	P04004<br>P01042	-1.3	0.041
33	Vitronectin<br>Kininogen-1<br>Lumican	8<br>8<br>20	P04004<br>P01042<br>P51884	-1.2	0.007
34	Vitronectin<br>Kininogen-1	8<br>8	P04004<br>P01042	-1.2	0.011
35	unidentified			-1.3	0.013
36	Fibrinogen gamma chain<br>Plasma protease C1 inhibitor<br>Hemopexin	32<br>8<br>12	P02679<br>P05155<br>P02790	1.5	0.003
38	unidentified			-1.4	0.016
39	Complement factor B<br>Complement C4-A; Complement C4-B	16<br>4; 4	P00751<br>P0C0L4; P0C0L5	1.6	0.006
40	Plasminogen	31	P00747	1.3	0.002
41	Fibrinogen beta chain<br>Plasminogen	30<br>25	P02675<br>P00747	1.3	0.008
42	Beta-2-glycoprotein 1<br>Complement C3	24<br>6	P02749<br>P01024	1.4	0.008
43	unidentified			1.8	0.005
45	Fibronectin<br>Fibrinogen gamma chain	10<br>22	P02751<br>P02679	1.4	0.005
46	Ceruloplasmin<br>Alpha-1B-glycoprotein<br>Inter-alpha-trypsin inhibitor heavy chain H2	14<br>22<br>5	P00450<br>P04217<br>P19823	1.4	0.005
47	unidentified			-1.3	0.022
48	unidentified			-1.3	0.016
49	Tetranectin	6	P05452	-1.3	0.029
50	Apolipoprotein A-I<br>Inter-alpha-trypsin inhibitor heavy chain H4<br>Protein AMBP	32<br>5<br>14	P02647<br>Q14624<br>P02760	1.2	0.023
51	Serum paraoxonase/arylesterase 1<br>Zinc-alpha-2-glycoprotein	23<br>9	P27169<br>P25311	-1.3	0.016
52	Apolipoprotein A-IV	12	P06727	-1.2	0.013
53	unidentified			1.2	0.042
54	Fibrinogen beta chain<br>Serum albumin<br>Antithrombin-III<br>Beta-2-glycoprotein 1	30<br>14<br>8<br>24	P02675<br>P02768<br>P01008<br>P02749	-2	0.019
55	Complement factor B<br>Plasma protease C1 inhibitor<br>Complement component C7	32<br>15<br>10	P00751<br>P05155<br>P10643	1.2	0.024
56	Complement factor B<br>Plasma protease C1 inhibitor	29<br>8	P00751<br>P05155	1.1	0.037
57	Complement C4-A; Complement C4-B<br>Inter-alpha-trypsin inhibitor heavy chain H4<br>Angiotensinogen<br>Haptoglobin<br>Prothrombin	8; 8<br>7<br>6<br>7<br>8	P0C0L4; P0C0L5<br>Q14624<br>P01019<br>P00738<br>P00734	1.3	0.031
58	Complement factor H<br>Alpha-2-macroglobulin	25<br>14	P08603<br>P01023	1.3	0.013
78	Serum albumin<br>Hemopexin	38<br>15	P02768<br>P02790	-2	0.019

ac accession number (Swiss-Prot), *sc %* protein sequence coverage

List of spots that significantly differ when comparing plasma proteomes of unstable angina pectoris patients with the control group

spot	protein	sc %	ac	fold	p
1	Serum amyloid A protein	52	P02735	2.5	0.005
2	unidentified			1.4	0.002
3	Serum amyloid A protein	52	P02735	2.2	0.006
4	Serum amyloid A protein	40	P02735	1.4	0.019
7	Transthyretin	55	P02766	-1.3	0.017
8	Transthyretin	39	P02766	-1.3	0.016
10	unidentified			1.4	0.007
13	Transthyretin	55	P02766	-1.5	0.004
14	Transthyretin	64	P02766	-1.2	0.018
16	Transthyretin<br>Apolipoprotein A-I	55<br>16	P02766<br>P02647	-1.7	0.003
17	Apolipoprotein A-I<br>Inter-alpha-trypsin inhibitor heavy chain H4<br>Apolipoprotein E	26<br>7<br>13	P02647<br>Q14624<br>P02649	-1.5	0.006
18	Protein AMBP	11	P02760	-1.4	0.002
19	Clusterin	27	P10909	-1.2	0.001
20	unidentified			-1.2	0.001
21	unidentified			-1.3	0.018
26	Fibrinogen beta chain<br>Fibrinogen alpha chain	50<br>6	P02675<br>P02671	1.2	0.038
27	Complement C3<br>Fibrinogen alpha chain<br>Fibrinogen beta chain	25<br>21<br>23	P01024<br>P02671<br>P02675	1.2	0.013
28	Serum albumin<br>Fibrinogen alpha chain<br>Hemopexin	33<br>7<br>19	P02768<br>P02671<br>P02790	-1.5	0.029
31	Alpha-2-antiplasmin<br>Complement component C9<br>Histidine-rich glycoprotein	25<br>4<br>8	P08697<br>P02748<br>P04196	-1.2	0.018
32	Vitronectin<br>Kininogen-1	8<br>8	P04004<br>P01042	-1.4	0.011
33	Vitronectin<br>Kininogen-1<br>Lumican	8<br>8<br>20	P04004<br>P01042<br>P51884	-1.3	0.0009
34	Vitronectin<br>Kininogen-1	8<br>8	P04004<br>P01042	-1.4	0.0001
35	unidentified			-1.4	0.0003
43	unidentified			1.9	0.024
61	Apolipoprotein A-I	45	P02647	-1.8	0.013
62	Apolipoprotein A-I	34	P02647	-1.5	0.031
63	Apolipoprotein A-I	32	P02647	-1.4	0.013
64	Apolipoprotein A-I	33	P02647	-1.7	0.017
65	Apolipoprotein A-I	33	P02647	-1.6	0.007
66	Apolipoprotein A-I	33	P02647	-1.6	0.008
67	Apolipoprotein A-I	31	P02647	-1.5	0.002
68	Apolipoprotein A-I	43	P02647	-1.2	0.021
69	Apolipoprotein A-I	16	P02647	-1.2	0.023
70	unidentified			1.5	0.019
71	unidentified			1.4	0.013
72	Serum amyloid A protein	49	P02735	1.3	0.028
73	unidentified			-1.2	0.005
74	Clusterin	11	P10909	-1.3	0.02
75	Kininogen-1<br>Lumican<br>Biotinidase<br>Vitronectin	11<br>12<br>4<br>5	P01042<br>P51884<br>P43251<br>P04004	-1.2	0.036
76	Complement C1s subcomponent<br>Cholinesterase<br>Apolipoprotein A-I	10<br>4<br>17	P09871<br>P06276<br>P02647	-1.2	0.035
77	Ceruloplasmin	9	P00450	-1.3	0.017

ac accession number (Swiss-Prot), *sc %* protein sequence coverage

List of spots that significantly differ when comparing plasma proteomes of stable angina pectoris patients with the control group

spot	protein	sc %	ac	fold	p
7	Transthyretin	55	P02766	-1.4	0.011
21	unidentified			-1.3	0.009
22	Fibrinogen gamma chain<br>Antithrombin-III<br>Vitamin D-binding protein<br>Apolipoprotein A-IV	43<br>12<br>13<br>16	P02679<br>P01008<br>P02774<br>P06727	1.2	0.009
42	Beta-2-glycoprotein 1<br>Complement C3	24<br>6	P02749<br>P01024	1.3	0.03
43	unidentified			1.6	0.01
44	Ceruloplasmin<br>Vitamin D-binding protein	26<br>27	P00450<br>P02774	-1.1	0.043
59	Apolipoprotein E	30	P02649	-1.2	0.024
60	unidentified			-1.3	0.038

ac accession number (Swiss-Prot), *sc %* protein sequence coverage
